# *KCNN4* is a diagnostic and prognostic biomarker that promotes papillary thyroid cancer progression

**DOI:** 10.18632/aging.103710

**Published:** 2020-08-28

**Authors:** Jialiang Wen, Bangyi Lin, Lizhi Lin, Yizuo Chen, Ouchen Wang

**Affiliations:** 1 Department of Thyroid and Breast Surgery, The First Affiliated Hospital of Wenzhou Medical University, Wenzhou, Zhejiang, People’s Republic of China

**Keywords:** KCNN4, biomarker, papillary thyroid cancer (PTC), epithelial-mesenchymal transition (EMT), apoptosis

## Abstract

The incidence of thyroid cancer remains high worldwide, and papillary thyroid cancer (PTC) is the most common type. Potassium Calcium-Activated Channel Subfamily N Member 4 (*KCNN4*) has been reported as an oncogene in various cancers. We examined expression of *KCNN4* in public databases and discovered that it is upregulated in PTC. We verified this finding using our own validated cohort and RNA sequencing data. We also found that *KCNN4* is a diagnostic and prognostic biomarker that is associated with disease-free survival, immune infiltration, and several other clinicopathological features of PTC. Gene Set Enrichment Analysis indicated that apoptotic and epithelial-mesenchymal transition gene sets are both upregulated in PTC patients with higher *KCNN4* levels. In PTC cell lines, silencing *KCNN4* inhibited cell proliferation, migration and invasion. Moreover, quantitative real-time PCR and Western blotting indicated that silencing *KCNN4* increased expression of apoptotic genes in PTC cells and reduced the expression of genes involved in their epithelial-mesenchymal transition. These results suggest that KCNN4 promotes PTC progression by inducing epithelial-mesenchymal transition and suppressing apoptosis, which suggests *KCNN4* may be a useful diagnostic and prognostic biomarker of PTC.

## INTRODUCTION

In recent years, the incidence of thyroid cancer (TC) has increased sharply at an annual rate of 3%. Thus, 40,170 new cases of TC are expected in the US in 2020, accounting for approximately 4% of all cancer cases in women [1, 2]. Papillary thyroid carcinoma (PTC) accounts for 83.6% of all TC cases [3]. PTC patients usually have a relatively good prognosis, with a long-term survival rate of 90-95% after surgery or radioiodine therapy; however, the subset of patients who develop local invasion or distant metastasis may be at greater risk of recurrence or death [3]. Lymph node metastasis (LNM) occurs in about 10-50% of PTC patients, and Wada et al. reported that the recurrence rates were 16.3% and 0% in patients with and without LNM, respectively [4, 5]. The overall survival rate of patients with local recurrence is about 70-85%, while the long-term survival rate of patients with distant metastasis is 30-60% [6–9]. Therefore, it is extremely important to identify biomarkers that can discriminate between high-risk and low-risk PTC patients.

The B-type Raf Kinase (*BRAF*) V600E mutation has been found in about 50% of PTC patients, particularly those with the aggressive subtype [10, 11]. In some studies, the *BRAF* V600E mutation has been associated with poorer clinicopathological outcomes of PTC [12, 13]; however, several studies have demonstrated that the *BRAF* V600E status alone is not an adequate prognostic biomarker of PTC [14–16]. The synergistic effects of *BRAF* V600E mutations and Telomerase Reverse Transcriptase promoter mutations are thought to generate more aggressive clinical characteristics and worse prognoses than the individual mutations in PTC patients [17, 18]. Mutations in *RAS*, *RET* and *PIK3CA* (Phosphatidylinositol-4,5-Bisphosphate 3-Kinase Catalytic Subunit Alpha) have also been observed in PTC [19, 20]. Based on genomic, epigenomic and proteomic profiling, PTC can be classified as BRAF^V600E^-like or RAS-like. BRAF^V600E^-like PTC (driven by BRAF^V600E^) cannot respond to negative feedback from Extracellular Signal-Regulated Kinase (ERK) to RAF, so Mitogen-Activated Protein Kinase (MAPK) signaling is hyperactivated. On the other hand, RAS-like PTC (driven by RAS and Receptor Tyrosine Kinase fusions) accepts feedback from ERK, and thus exhibits aberrant activation of Phosphoinositide 3-Kinase signaling and lower MAPK activity than BRAF^V600E^-like PTC [21]. Despite tremendous progress in TC research, many molecular characteristics of TC are not well understood, so further study on the tumorigenesis and progression of TC is needed.

Ion channels of the plasma membrane are known to be involved in various cancers [22]. For example, Potassium Voltage-Gated Channel Subfamily H Member 1 is associated with a poor prognosis in ovarian cancer [23], and Piezo Type Mechanosensitive Ion Channel Component 1 promotes prostate cancer proliferation by activating the Akt/Mammalian Target of Rapamycin pathway [24]. Regarding thyroid cancer, Voltage Dependent Anion Channel 2 improves the viability of PTC cell lines [25], whereas Sodium Voltage-Gated Channel Beta Subunit 4 is an independent indicator of enhanced recurrence-free survival in PTC patients [26]. Thus, ion channels may have essential functions in PTC.

The Potassium Calcium-Activated Channel Subfamily N (KCNN) family includes four proteins (KCNN1-KCNN4) that constitute a heterotetrameric voltage-independent potassium channel. The KCNNs are involved in various cancers: low *KCNN3* expression promotes drug resistance and may predict a poor prognosis in ovarian cancer [27], high *KCNN4* expression is associated with poor survival in pancreatic cancer patients [28], and *KCNN2* is overexpressed in proximal tumors compared to distal tumors in colorectal cancer [29]. However, the involvement of the KCNN family in TC is still unknown.

In the present study, we examined *KCNN4* expression in PTC tissues using various online datasets and our own locally validated data. We also analyzed the diagnostic and prognostic value of *KCNN4* expression and its relationship with the clinicopathological features of PTC. Finally, we performed loss-of-function assays to explore the function of KCNN4 in PTC cell lines. Our work suggests that KCNN4 may be an essential biomarker and tumor promoter in PTC.

## RESULTS

### *KCNN4* is significantly upregulated in PTC

**Figure 1:**
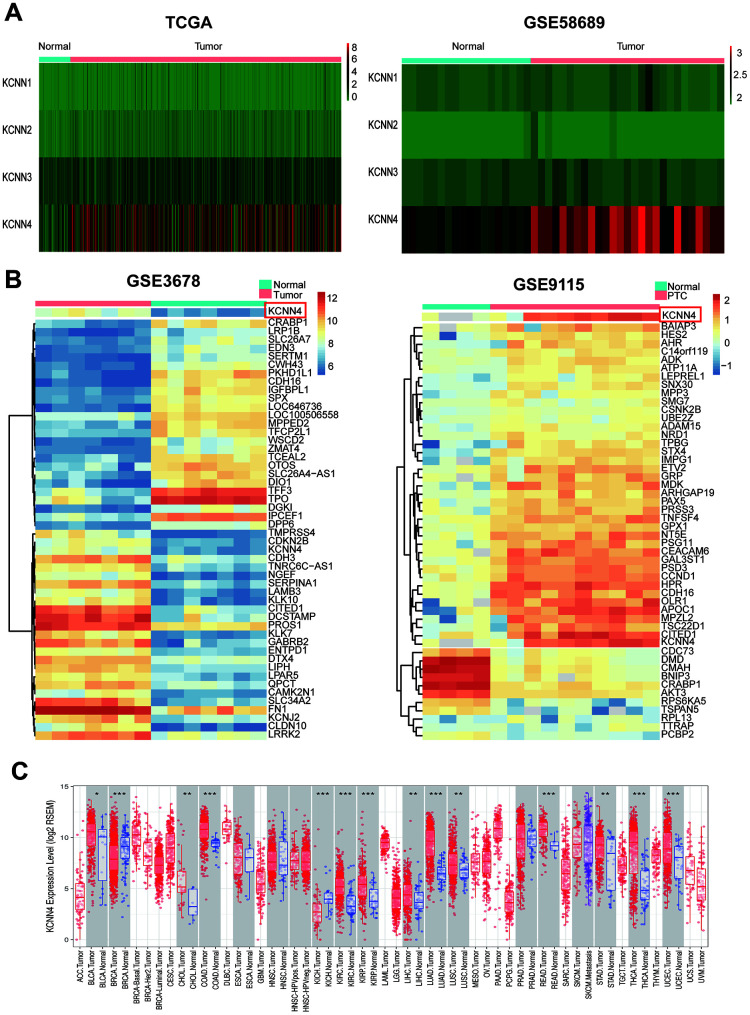
***KCNN4* was differentially expressed in PTC and most other cancers.** (**A**) The expression of the KCNN family in PTC was assessed using data from TCGA and GSE58689. (**B**) Heatmap of the top 50 significantly differentially expressed genes in GSE3678 and GSE9115, based on the adjusted p-value. (**C**) *KCNN4* was overexpressed in multiple cancers in TCGA. The statistical significance of differential expression was evaluated using the Wilcoxon test. *p<0.05, **p<0.01, ***p<0.001. ACC: Adrenocortical carcinoma; BLCA: Bladder urothelial carcinoma; BRCA: Breast invasive carcinoma; CESC: Cervical squamous cell carcinoma and endocervical adenocarcinoma; CHOL: Cholangiocarcinoma; COAD: Colon adenocarcinoma; DLBC: Lymphoid neoplasm diffuse large B-cell lymphoma; ESCA: Esophageal carcinoma; GBM: Glioblastoma multiforme; HNSC: Head and neck squamous cell carcinoma; KICH: Kidney chromophobe; KIRC: Kidney renal clear cell carcinoma; KIRP: Kidney renal papillary cell carcinoma; LAML: Acute myeloid leukemia; LGG: Brain lower grade glioma; LIHC: Liver hepatocellular carcinoma; LUAD: Lung adenocarcinoma; LUSC: Lung squamous cell carcinoma; MESO: Mesothelioma; OV: Ovarian serous cystadenocarcinoma; PAAD: Pancreatic adenocarcinoma; PCPG: Pheochromocytoma and paraganglioma; PRAD: Prostate adenocarcinoma; READ: Rectum adenocarcinoma; SARC: Sarcoma; SKCM: Skin cutaneous melanoma; STAD: Stomach adenocarcinoma; TGCT: Testicular germ cell tumor; THCA: Thyroid carcinoma; THYM: Thymoma; UCEC: Uterine corpus endometrial carcinoma; UCS: Uterine carcinosarcoma; UVM: Uveal melanoma.

To analyze the expression of the four KCNN family members in PTC, we downloaded transcriptome sequencing data and microarray data from The Cancer Genome Atlas (TCGA) and GSE58689, respectively. Only *KCNN4* was significantly overexpressed in tumor tissues compared to nontumorous thyroid tissues (Figure 1A). Furthermore, we mined the differentially expressed genes between PTC tissues and adjacent normal tissues in the GSE3678 and GSE9115 datasets. After data normalization, 90 and 100 differentially expressed genes were extracted from GSE3678 and GSE9115, respectively (adjusted p-value < 0.05 and log2|fold change| ≥ 2). As shown in the heatmap (Figure 1B), *KCNN4* was markedly upregulated in PTC tissues among the top 50 significantly differentially expressed genes in both datasets, based on the adjusted p-value (GSE3678: *KCNN4*, log2|fold change|=2.30, false discovery rate [FDR]=8.40×10^-5^; GSE9115: *KCNN4*, log2|fold change|=3.73, FDR=0.01). Next, the Tumor IMmune Estimation Resource (TIMER) database was used to perform a pan-cancer analysis of *KCNN4* expression. The results indicated that *KCNN4* was upregulated in various cancers in TCGA (Figure 1C).

**Figure 2:**
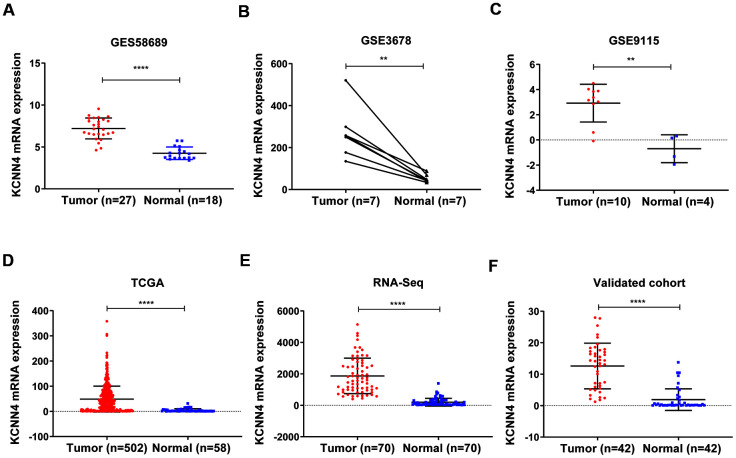
***KCNN4* was overexpressed in PTC.** (**A**–**D**) *KCNN4* mRNA levels were greater in PTC tissues than in normal thyroid tissues in GSE58689, GSE3678, GSE9115 and TCGA. (**E**) *KCNN4* was upregulated in 70 PTC tissues compared with matched adjacent nontumorous thyroid tissues from our RNA sequencing dataset. (**F**) The overexpression of *KCNN4* in PTC was verified in our validated cohort using qRT-PCR. Statistical analyses were performed as follows: (**A**, **C**, **D**): Mann-Whitney test; (**B**): Paired t-test; (**E**, **F**): Wilcoxon test. **p<0.01, ****p<0.0001.

The mRNA levels of *KCNN4* in TC tissues and adjacent nontumorous tissues in the aforementioned databases are shown in Figure 2A–2D (GSE58689: PTC tissues 7.21 ± 1.24, Normal tissues 4.25 ± 0.74, *p*<0.0001; GSE3678: PTC tissues 270.8 ± 123.5, Normal tissues 52.50 ± 19.48, *p*<0.01; GSE9155: PTC tissues 2.92 ± 1.50, Normal tissues -0.70 ± 1.11, *p*<0.01; TCGA: PTC tissues 48.82 ± 51.50, Normal tissues 4.44 ± 6.13, *p*<0.0001). We also performed RNA sequencing on 70 pairs of PTC tissues and matched adjacent nontumorous tissues, and observed the same trend (Figure 2E; PTC tissues 1868 ± 1127, Normal tissues 197.3 ± 245.3, *p*<0.0001). Furthermore, we used quantitative real-time PCR (qRT-PCR) to analyze *KCNN4* expression in 42 pairs of PTC tissues and matched normal thyroid tissues (validated cohort). As expected, *KCNN4* mRNA levels were significantly greater in PTC tissues than in normal thyroid tissues (Figure 2F; PTC tissues 12.59 ± 7.26, Normal tissues 1.90 ± 3.44, *p*<0.0001). These findings demonstrated that *KCNN4* was overexpressed in PTC tissues.

### *KCNN4* overexpression is associated with a range of clinicopathological factors in PTC

Next, we evaluated the correlation between *KCNN4* expression and clinical features of PTC in TCGA and our validated cohort. Patients were split into two groups based on the median *KCNN4* level. In the cohort from TCGA, higher *KCNN4* expression was associated with a higher T stage (*p=*0.001), a greater incidence of LNM (*p*<0.0001), a more advanced disease stage (*p=*0.001) and a higher frequency of the classical type (*p*<0.0001) (Table 1). In our validated cohort, *KCNN4* expression also correlated significantly with the tumor size (*p=*0.028), LNM (*p*<0.030) and disease stage (*p=*0.009) (Table 2).

**Table 1:** Correlation between KCNN4 expression and clinicopathologic factors in the TCGA cohort.

**Clinicopathologic factors**	**Patients**	**High expression**	**Low expression**	**p-value**
Gender				
Female	367	180	187	0.546
Male	135	71	64	
Age (years)				
<60	389	196	193	0.831
≥60	113	55	58	
Histological type				
Classical	356	203	153	<0.0001*
Other types	146	48	98	
Neoplasm focus type				
Unifocal	266	135	131	0.928
Multifocal	226	113	113	
T stage				
I+II	307	135	172	0.001*
III+IV	193	114	79	
Lymph node metastasis			
Yes	222	150	72	<0.0001*
No	229	83	146	
Disease stage(AJCC7)				
I+II	333	149	184	0.001*
III+IV	167	101	66	
New event				
Yes	44	27	17	0.155
No	458	224	234	

Note: *p-value< 0.05.

Abbreviations: KCNN4, Potassium calcium-activated channel subfamily N member 4; TCGA, The Cancer Genome Atlas; AJCC7, American Joint Committee on Cancer 7^th^ edition.

**Table 2:** Correlation between KCNN4 expression and clinicopathologic factors in the validated cohort.

**Clinicopathologic factors**	**Patients**	**High expression**	**Low expression**	**p-value**
Gender				
Female	23	13	10	0.352
Male	19	8	11	
Age (years)				
≥55	15	8	7	0.747
<55	27	13	14	
Tumor size (mm)				
≥10	25	16	9	0.028*
<10	17	5	12	
Lymph node metastasis				
Yes	23	15	8	0.030*
No	19	6	13	
Neoplasm focus type				
Multifocal	8	3	5	0.697
Unifocal	34	18	16	
Disease stage(AJCC7)				
III+IV	14	11	3	0.009*
I+II	24	10	18	

Note: *p-value< 0.05.

Abbreviations: KCNN4, Potassium calcium-activated channel subfamily N member 4; AJCC7, American Joint Committee on Cancer 7^th^ edition.

**Figure 3:**
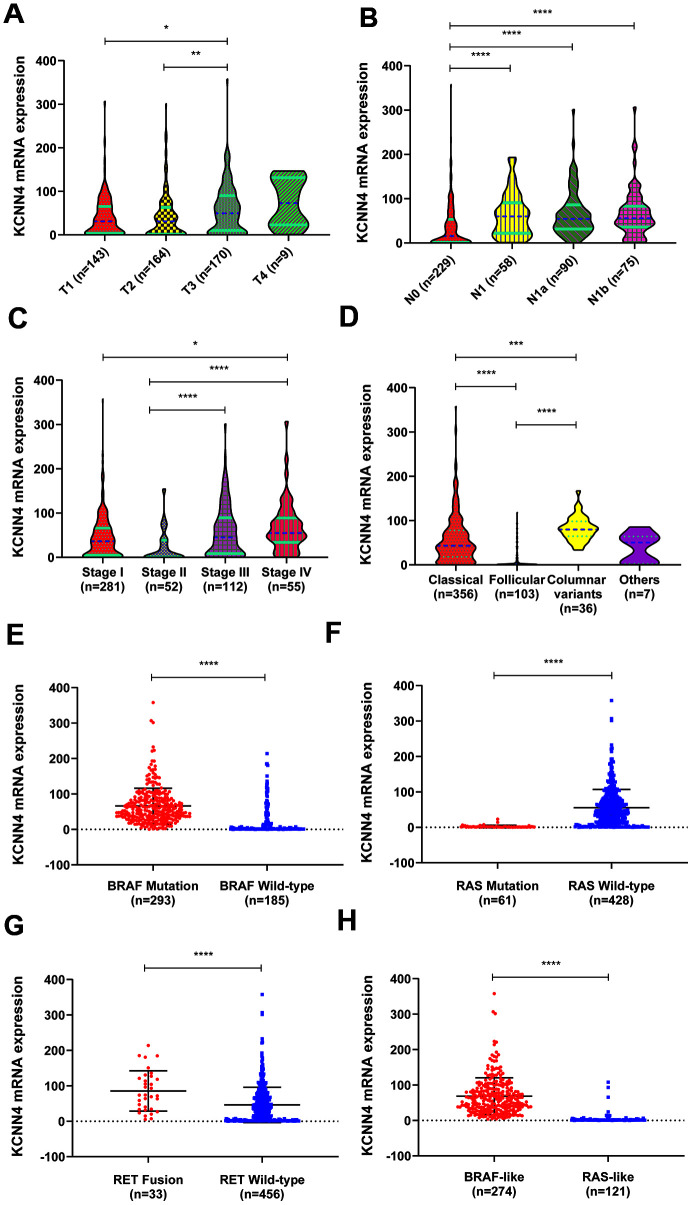
**The relationship between *KCNN4* expression and clinicopathological characteristics of PTC.** (**A**–**C**) *KCNN4* expression varied according to the T stage, N stage and tumor stage. Higher *KCNN4* expression tended to be associated with worse clinicopathological features. (**D**) The expression of *KCNN4* in different subtypes of PTC. *KCNN4* was significantly upregulated in the columnar variant subtype and the classical subtype compared to the follicular subtype. (**E**) *KCNN4* expression was significantly higher in the *BRAF* mutation group than in the *BRAF* wild-type group. (**F**) *KCNN4* expression was significantly higher in the *RAS* wild-type group than in the *RAS* mutation group. (**G**) *KCNN4* expression was significantly higher in the *RET* fusion group than in the *RET* wild-type group. (**H**) *KCNN4* expression was higher in the BRAF-like group than in the RAS-like group. Statistical analyses were performed as follows: A-D: Kruskal-Wallis test; E-H: Mann-Whitney test. *p<0.05, **p<0.01, ***p<0.001, ****p<0.0001.

Then we evaluated *KCNN4* expression in PTC patients with different tumor grades, stages, subtypes and mutations in TCGA. As illustrated in Figure 3A–3C, most patients with higher T stages, N stages and disease stages had higher mRNA levels of *KCNN4*. In a subtype analysis, *KCNN4* levels were significantly greater in the classical and columnar variant subtypes than in the follicular subtype (Figure 3D). We also divided patients according to their driver mutation status, and found that *KCNN4* expression was significantly greater in the *BRAF* mutation group (Figure 3E, *p*<0.0001), the *RAS* wild-type group (Figure 3F, *p*<0.0001) and the *RET* fusion group (Figure 3G, *p*<0.0001) than in their respective counterpart groups. *KCNN4* expression was also significantly greater in the BRAF-like group than in the RAS-like group (Figure 3H; BRAF-like: 68.63 ± 51.57, RAS-like: 4.36 ± 14.22, *p*<0.0001). Thus, *KCNN4* expression was associated with many clinicopathological features of PTC.

### The upregulation of *KCNN4* increases the risk of LNM in PTC

To examine whether high *KCNN4* expression is a major risk factor for LNM in PTC, we constructed regression models using data from TCGA (Table 3). Univariate logistic regression demonstrated that high *KCNN4* expression (odds ratio [OR]=3.665, 95% confidence interval [CI]: 2.483-5.409, *p*<0.001), female (OR=0.640, 95% CI: 0.422-0.972, *p*=0.036), disease stage III/IV (OR=3.524, 95% CI: 2.336-5.316, *p*<0.001), T stage III/IV (OR=2.688, 95% CI: 1.830-3.970, *p*<0.001) and classical type (OR=2.370, 95% CI: 1.535-3.660, *p*<0.001) were associated with the risk of LNM. Multivariate logistic regression indicated that high *KCNN4* expression (OR=2.914, 95% CI: 1.925-4.411, *p*<0.001), disease stage III/IV (OR=2.708, 95% CI: 1.656-4.428, *p*<0.001), T stage III/IV (OR=1.703, 95% CI: 1.054-2.753, *p*=0.030) and classical type (OR=2.506, 95% CI: 1.528-4.110, *p*<0.001) were associated with the risk of LNM, whereas gender was excluded from the model (OR=0.770, 95% CI: 0.482-1.229, *p*=0.274). These data indicated that elevated *KCNN4* expression increased the risk of LNM.

**Table 3:** Univariate and multivariate logistic regression for the risk of lymph node metastasis in the TCGA cohort.

**Factors**	**Univariate analysis**				**Multivariate analysis**		
	**OR**	**95% CI**	**p-value**		**OR**	**95% CI**	**p-value**
KCNN4 expression (high *vs.* low)	3.665	2.483-5.409	<0.001*		2.914	1.925-4.411	<0.001*
Age (>60 *vs.* <60)	0.653	0.416-1.024	0.063		-		
Gender (female *vs.* male)	0.640	0.422-0.972	0.036*		0.770	0.482-1.229	0.274
Disease stage (III,IV *vs.* I,II)	3.524	2.336-5.316	<0.001*		2.708	1.656-4.428	<0.001*
T stage (III,IV *vs.* I,II)	2.688	1.820-3.970	<0.001*		1.703	1.054-2.753	0.03*
New event	1.893	0.974-3.678	0.060		-		
Neoplasm focus type (Mul *vs.* Uni)	1.433	0.985-2.086	0.060		-		
Histological type (classical vs. others)	2.370	1.535-3.660	<0.001*		2.506	1.528-4.110	<0.001*

Note: *p-value< 0.05.

Abbreviations: KCNN4, Potassium calcium-activated channel subfamily N member 4; TCGA, The Cancer Genome Atlas; OR, Odds ratio; CI, Confidence interval.

### The diagnostic and prognostic value of *KCNN4* in PTC patients

**Figure 4:**
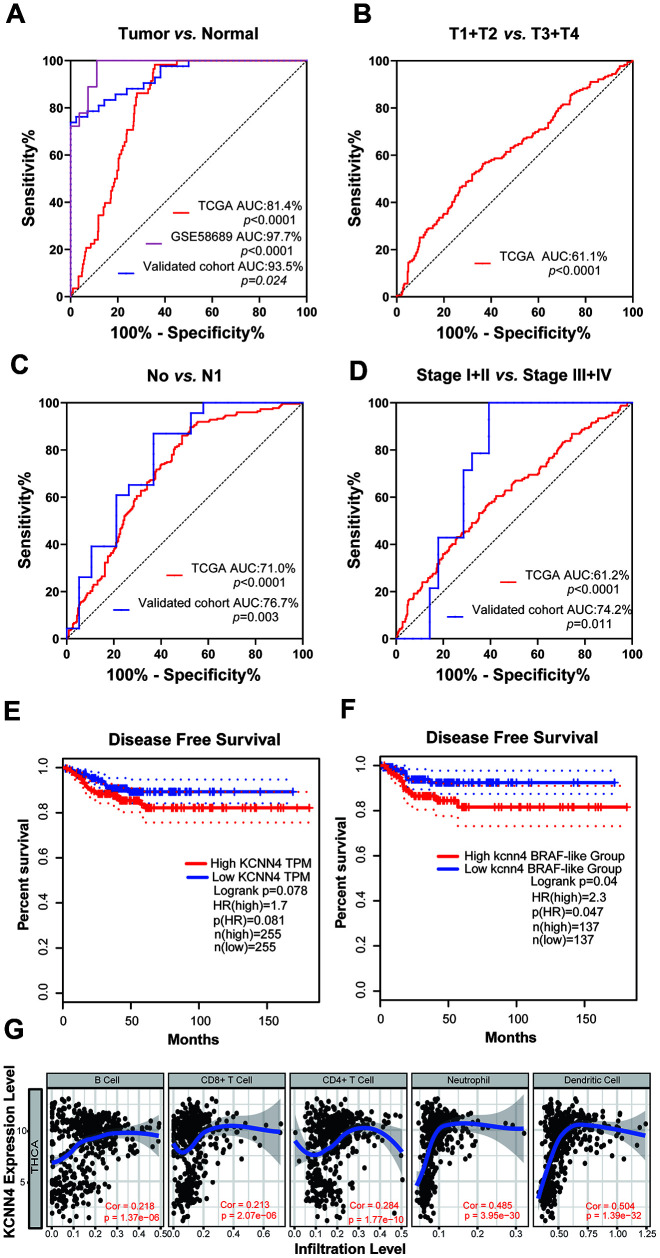
**The upregulation of *KCNN4* served as a prognostic and diagnostic biomarker in PTC.** (**A**) ROC curve analysis demonstrated that *KCNN4* could distinguish PTC from nontumorous tissues in TCGA, GSE58689 and our validated cohort. (**B**) Diagnostic value of *KCNN4* expression for the T stage in TCGA. (**C**, **D**) ROC curve analysis depicting *KCNN4* expression against the N stage and tumor stage in TCGA and our validated cohort. (**E**, **F**) Kaplan-Meier analyses of disease-free survival based on low and high *KCNN4* expression in PTC patients and the BRAF-like group from TCGA. (**G**) *KCNN4* expression was associated with immune infiltration in TCGA. THCA: Thyroid carcinoma; Cor: Correlation.

Next, we used receiver operating characteristic (ROC) curves to examine the diagnostic significance of *KCNN4* expression in TCGA, GSE58689 and our validated cohort. *KCNN4* expression distinguished between PTC tissues and normal tissues with an area under the ROC curve (AUC) of 81.4% (95% CI: 77.6-85.2%, *p*<0.0001) in TCGA, 93.5% (95% CI: 88.7-98.3%, *p*=0.024) in our validated cohort and 97.7% (95% CI: 94.4-100%, *p*<0.0001) in GSE58689 (Figure 4A). In TCGA, *KCNN4* also had diagnostic value for the T stage, with an AUC of 61.1% (95% CI: 55.9-66.3%, *p*<0.0001) (Figure 4B). For LNM, the AUC was 71.0% (95% CI: 66.2-75.8%, *p*<0.0001) in TCGA and 76.7% (95% CI: 61.7-91.7%, *p*=0.003) in our validated cohort (Figure 4C). The AUC for distinguishing tumor stage (I + II) / (III + IV) was 61.2% (95% CI: 56.0-66.5%, *p*<0.0001) in TCGA and 74.2% (95% CI: 59.4-89.1%, *p=*0.011) in our validated cohort (Figure 4D). These findings suggested that *KCNN4* expression may have diagnostic value for PTC patients.

To investigate the prognostic value of *KCNN4*, we performed a disease-free survival analysis using Kaplan-Meier curves from the Gene Expression Profiling Interactive Analysis (GEPIA) server. Higher *KCNN4* expression was associated with a greater risk of relapse or death (hazard ratio [HR]=1.7, *p*=0.078), especially in the BRAF-like subgroup (HR=2.3, *p*=0.047) (Figure 4E, 4F). Thus, *KCNN4* overexpression may predict a worse prognosis in PTC patients.

### *KCNN4* overexpression is associated with immune cell infiltration

Previous studies have indicated that KCNN4 can alter antigen presentation and various immune cell functions [30, 31]. Using TCGA data from the TIMER online tool, we explored the association between *KCNN4* expression and immune cell infiltration in PTC. *KCNN4* expression correlated positively with the levels of infiltrating B cells (Correlation=0.218, *p*<0.0001), CD8+ T cells (Correlation=0.213, *p*<0.0001), CD4+ T cells (Correlation=0.284, *p*<0.0001), neutrophils (Correlation=0.485, *p*<0.0001) and dendrites (Correlation=0.504, *p*<0.0001) (Figure 4G).

### *KCNN4* is overexpressed in PTC cell lines and promotes PTC cell proliferation

**Figure 5:**
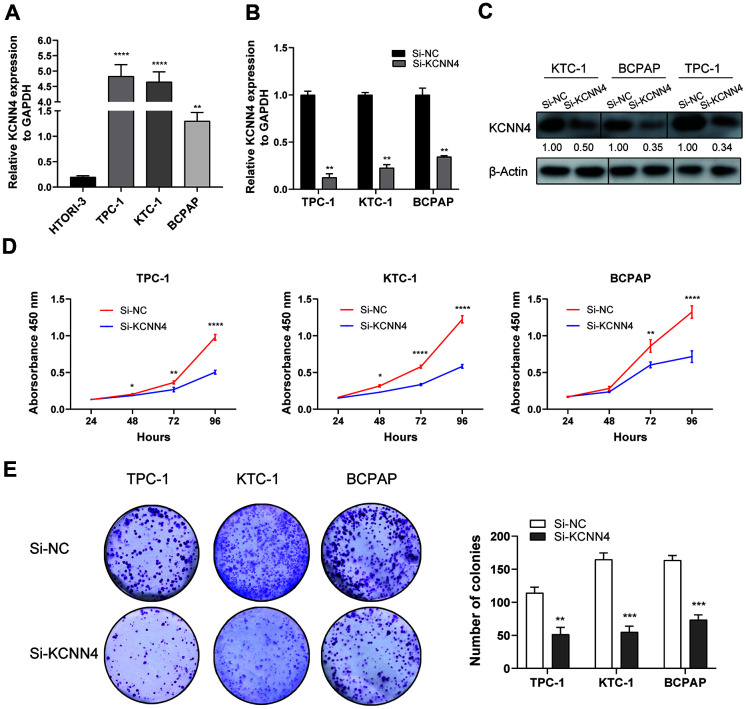
***KCNN4* was upregulated in PTC cell lines and promoted their proliferation *in vitro*.** (**A**) The relative expression of *KCNN4* in PTC cell lines (compared with *GAPDH*). *KCNN4* was upregulated in three PTC cell lines (KTC-1, TPC-1 and BCPAP) compared to the normal thyroid cell line HTORI-3. (**B**) *KCNN4* expression was lower in the Si-KCNN4 group than in the Si-NC group in the three PTC cell lines. (**C**) KCNN4 protein expression was lower in the Si-KCNN4 group than in the Si-NC group. (**D**, **E**) CCK-8 assays and colony formation assays in the three PTC cell lines. Two-way analysis of variance was used to analyze the CCK-8 assay data, and Student’s t-test was used for the others. *p<0.05, **p<0.01, ***p<0.001, ****p<0.0001.

We then examined the mRNA levels of *KCNN4* in PTC and normal thyroid cell lines using qRT-PCR. *KCNN4* expression was markedly higher in TPC-1 (4.83 ± 0.375, *p*<0.0001), KTC-1 (4.65 ± 0.327, *p*<0.0001) and BCPAP (1.30 ± 0.168, *p*<0.01) cells than in normal HTORI-3 cells (0.198 ± 0.025) (Figure 5A). Next, we used small interfering RNA (siRNA) to knock down *KCNN4* (Si-KCNN4) in PTC cells. The siRNA reduced *KCNN4* mRNA levels by > 50% in all three PTC cell lines (Figure 5B, *p*<0.01), and also significantly reduced KCNN4 protein levels (Figure 5C).

Next, we assessed the effects of KCNN4 on PTC cell proliferation. A Cell Counting Kit-8 (CCK-8) assay demonstrated that knocking down *KCNN4* reduced the proliferative capacity of PTC cells, particularly in the last 24 hours of measurement (Figure 5D, *p*<0.0001). Additionally, a colony formation assay indicated that the downregulation of *KCNN4* attenuated the proliferative abilities of TPC-1 cells (negative control siRNA [Si-NC]: 114.3 ± 8.5 colonies, Si-KCNN4: 51.7 ± 10.4 colonies, *p*<0.01), KTC-1 cells (Si-NC: 165.0 ± 9.6 colonies, Si-KCNN4: 55.0 ± 8.9 colonies, *p*<0.001) and BCPAP cells (Si-NC: 163.7 ± 7.0 colonies, Si-KCNN4: 73.7 ± 7.4 colonies, *p*<0.001) (Figure 5E). Thus, the downregulation of *KCNN4* inhibited the proliferation of PTC cell lines.

### *KCNN4* knockdown reduces the migration and invasion capacities of PTC cells

**Figure 6:**
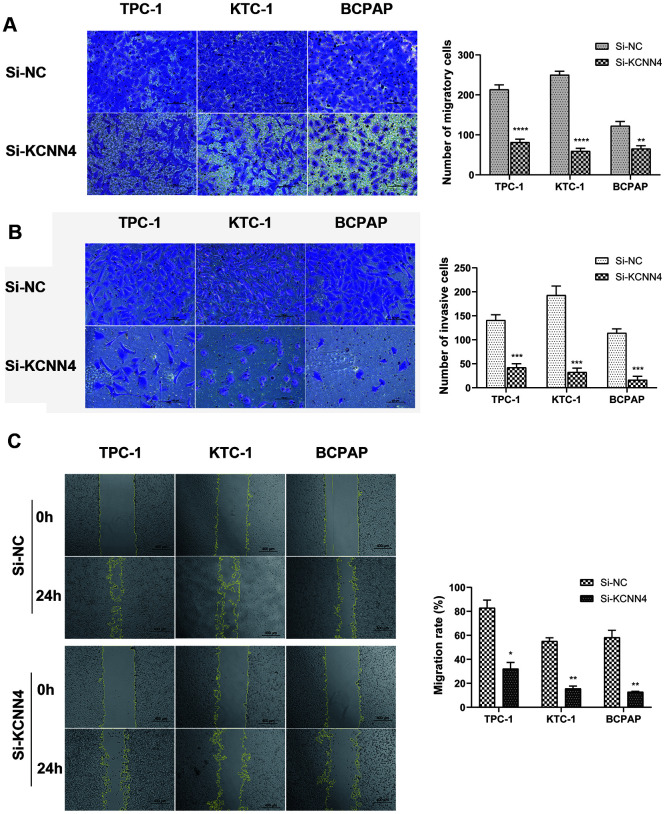
**Downregulation of *KCNN4* inhibited the migration and invasion of PTC cell lines.** (**A**, **B**) Transwell migration and Matrigel invasion assays in the *KCNN4*-knockdown group and the corresponding control group in three PTC cell lines. The quantitative results of the migration and invasion assays were determined from five random fields. (**C**) A wound-healing assay indicated that downregulating *KCNN4* reduced the migration abilities of PTC cell lines. The data came from at least two independent experiments. *p<0.05, **p<0.01, ***p<0.001, ****p<0.0001 in comparison with the Si-NC group using Student’s t-test.

Since *KCNN4* expression correlated with LNM in PTC, we performed Transwell migration assays, wound healing assays and Matrigel invasion assays to assess the impact of KCNN4 on PTC cell migration and invasion. The migration and invasion abilities of TPC-1, KTC-1 and BCPAP cells transfected with Si-KCNN4 were significantly lower than those of cells transfected with Si-NC (Figure 6A, 6B). The number of migratory cells was as follows - TPC-1: Si-NC 213.7 ± 11.4, Si-KCNN4 81.7 ± 7.4, *p*<0.0001; KTC-1: Si-NC 250.0 ± 9.0, Si-KCNN4 59.7± 6.8, *p*<0.0001; BCPAP: Si-NC 122.7 ± 11.1, Si-KCNN4 65.7 ± 7.1, *p*<0.01. The number of invading cells was - TPC-1: Si-NC 141.0 ± 11.1, Si-KCNN4 42.3 ± 7.8, *p*<0.001; KTC-1: Si-NC 193.0 ± 19.1, Si-KCNN4 32.7 ± 8.3, *p*<0.001; BCPAP: Si-NC 114.3 ± 8.5, Si-KCNN4 16.3 ± 7.5, *p*<0.001. In the wound healing assay, 24 hours after the scratch, the migration rate was reduced to less than 40% of the control value when *KCNN4* was downregulated (Figure 6C). These data indicated that KCNN4 may promote tumor metastasis in PTC.

### KCNN4 suppresses apoptosis and induces the epithelial-mesenchymal transition (EMT) in thyroid cancer cell lines

**Figure 7:**
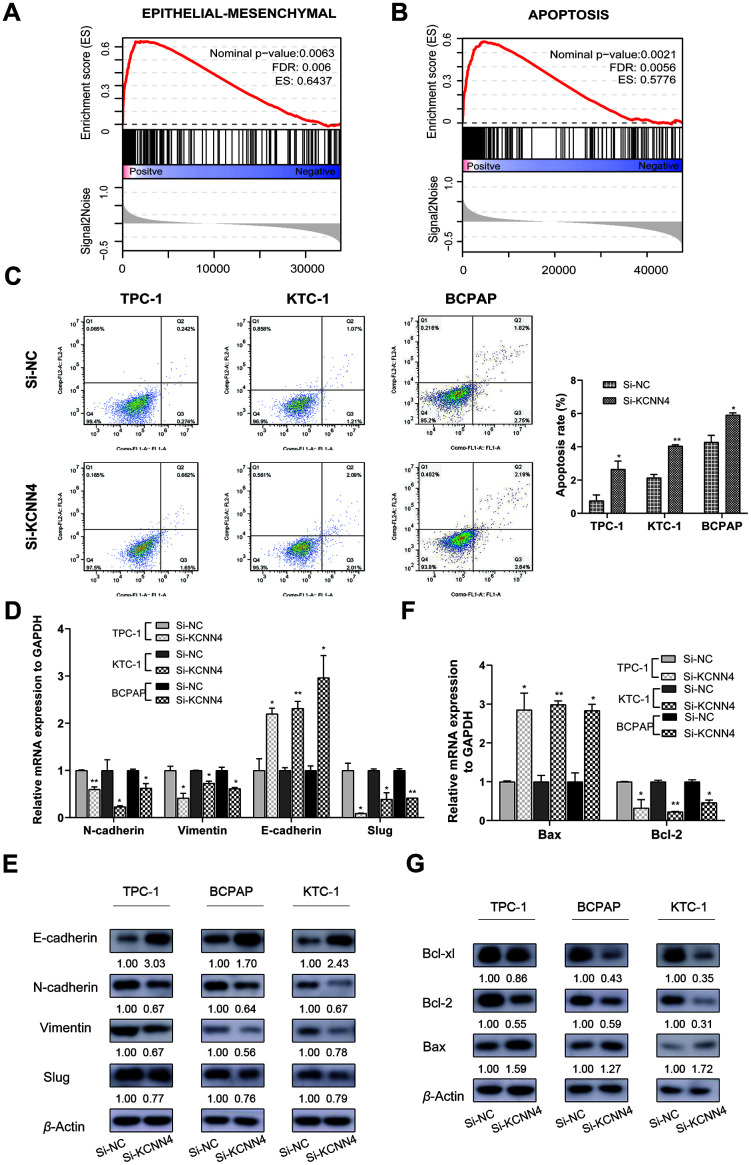
**The downregulation of *KCNN4* prevented the EMT while promoting apoptosis in PTC cell lines.** (**A**, **B**) Single GSEA based on data from TCGA demonstrated that *KCNN4* was associated with the EMT and apoptosis. (**C**) Silencing *KCNN4* promoted apoptosis in PTC cell lines. (**D**, **E**) Silencing *KCNN4* upregulated E-cadherin and downregulated N-cadherin, Vimentin and Slug at the mRNA and protein levels. (**F**) qRT-PCR demonstrated that downregulating *KCNN4* enhanced *Bax* expression but reduced *Bcl-2* expression. (**G**) Western blotting assays revealed that downregulating *KCNN4* increased Bax expression and reduced Bcl-2 and Bcl-xl expression at the protein level. *p<0.05 and **p<0.01 in comparison with the Si-NC group using Student’s t-test.

Single Gene Set Enrichment Analysis (GSEA) was used to identify genes that may be regulated by KCNN4 in PTC. Based on the median level of *KCNN4*, PTC patients from TCGA were divided into two groups. We discovered that genes involved in the EMT (FDR=0.006, Enrichment score=0.6437) and apoptosis (FDR=0.0056, Enrichment score=0.5776) were associated with *KCNN4* expression (Figure 7A, 7B). Therefore, we used flow cytometry to detect apoptosis in PTC cell lines after the downregulation of *KCNN4*. The apoptosis rate, calculated based on the total percentage of cells in the second and third quadrants, was higher in the *KCNN4*-knockdown group than in the control group in each cell line (TPC-1: Si-NC 0.76 ± 0.34, Si-KCNN4 2.66 ± 0.49, *p*<0.05; KTC-1: Si-NC 2.14 ± 0.20, Si-KCNN4 4.05± 0.07, *p*<0.01; BCPAP: Si-NC 4.29 ± 0.40, Si-KCNN4 5.92 ± 0.12, *p*<0.05) (Figure 7C).

Next, we quantified the mRNA and protein levels of several representative markers of the EMT and apoptosis in PTC cells. E-cadherin is an epithelial marker that suppresses tumor invasion, and its expression is negatively regulated by transcription factors such as Slug and Snail1 [32, 33]. Mesenchymal markers including Vimentin and N-cadherin are indicators of the EMT and cancer metastasis [34, 35]. The switch from E-cadherin to N-cadherin is an important hallmark of EMT induction [36]. We found that the downregulation of *KCNN4* reduced N-cadherin, Vimentin and Slug expression while elevating E-cadherin expression (Figure 7D, 7E).

The Bcl-2 gene family, which participates in mitochondrion-induced cellular apoptosis, includes both anti-apoptotic (Bcl-2 and Bcl-xl) and pro-apoptotic genes (Bax). Bax exerts pro-apoptotic effects by sequestering Bcl-2 or Bcl-xl in the form of a heterodimer [37]. In our study, the downregulation of *KCNN4* enhanced Bax expression and reduced Bcl-2 and Bcl-xl expression (Figure 7F, 7G). These results demonstrated that KCNN4 promotes the EMT and hinders apoptosis in PTC.

## DISCUSSION

TC has the fifth highest incidence rate of all cancers in women, following breast, lung, colorectal and uterine cancer [2]. Although patients with low-risk TC have a relatively good prognosis, those who experience recurrence or distant metastasis have a higher risk of death. Therefore, identifying high-risk patients is a crucial task for clinicians. To this end, it is indispensable to delineate the mechanisms of PTC and further mine the biomarkers for risk stratification. Numerous studies have demonstrated that membrane ion channels regulate cell proliferation, apoptosis and migration in various cancers, and thus can serve as prognostic biomarkers and therapeutic targets [38–40]. There is much evidence about the vital functions of ion channels in PTC, as well [26, 41].

*KCNN4*, a member of the KCNN family, encodes a potassium ion channel activated by calcium influx. The cancer-promoting effects of KCNN4 have been observed in various cancers. For example, *KCNN4* is highly expressed in triple-negative breast cancer and was found to promote the migration and EMT of triple-negative breast cancer cells [42]. In hepatocellular carcinoma, *KCNN4* promoted invasion and metastasis *in vitro* by inducing the MAPK/ERK and EMT pathways [43, 44]. Rabjerg et al. reported that high *KCNN4* expression was associated with poor survival and a high metastasis potential in clear cell renal carcinoma [45]. However, the involvement of KCNN4 in TC has not been known.

In the present study, we first found that *KCNN4* was highly upregulated in PTC tissues compared to normal thyroid tissues in public databases. We then examined *KCNN4* mRNA levels using our own RNA sequencing data and a validated cohort including 42 matched PTC and adjacent normal tissues. The results were consistent with those of the bioinformatics analysis. Previous studies have demonstrated that high KCNN4 expression is associated with malignant features and poor prognoses in various cancers [28, 45]. When we investigated the correlation between *KCNN4* expression and clinicopathological factors in PTC, we found that patients with higher *KCNN4* levels had a higher T stage, a greater incidence of LNM and more advanced disease stages than patients with lower *KCNN4* levels. The expression of *KCNN4* varied among patients with different PTC subtypes and driver mutation statuses, as it was elevated in patients with the columnar variant and classical types, as well as in those with *BRAF* mutations, *RET* fusion and wild-type *RAS*. Regression analysis indicated that *KCNN4* overexpression was an independent predictor of LNM in PTC. In addition, ROC curve analysis indicated that *KCNN4* expression could distinguish PTC tissues from normal tissues. Patients with higher *KCNN4* levels also had worse disease-free survival than those with lower levels, and this effect was especially pronounced in the BRAF-like group, whose *KCNN4* levels were significantly greater than those of the RAS-like group. These results suggested that *KCNN4* is a potential biomarker for PTC.

Interestingly, we found a positive association between *KCNN4* expression and immune infiltration in PTC patients in TCGA. This finding was consistent with previous data indicating that KCNN4 enhance migration capacity of dendritic cells and positively regulate antigen presentation [46]. Chimote et al. reported that activation of KCNN4 channel could improve immune surveillance and the response to immune therapies in cancer [31]. It has also been reported that KCNN4 blockers can ameliorate autoimmune disorders and inflammation [47]. Some studies have indicated that LNM is more common in PTC patients with thyroid autoimmunity than in those without [48–50]. Thus, we speculate that KCNN4 may be a valuable therapeutic target for PTC patients with coexistent thyroid autoimmunity. However, further experiments are needed to confirm this hypothesis.

Our loss-of-function study demonstrated that KCNN4 promotes proliferation, migration and invasion while inhibiting apoptosis in PTC cell lines. GSEA demonstrated the statistically significant enrichment of gene signatures associated with apoptosis and EMT. Extensive studies have shown that the EMT is associated with tumor initiation, progression, stemness, migration and resistance in various cancers [35]. Vasko et al. reported that the EMT was a common phenomenon in PTC metastasis, and that Vimentin promoted the EMT in thyroid cancer cell lines [51]. Another hallmark of cancer cells is their evasion of apoptosis [52]. Certain transcription factors and other proteins involved in the EMT have been reported to facilitate tumor survival by hindering apoptosis [53]. For example, proteins in the Snail family not only induce the EMT, but also promote survival and cell movement [54]. On the other hand, Wu and Tang reported that Bcl-2, an anti-apoptotic protein, is also an important inducer of the EMT [55]. Thus, there seems to be crosstalk between the EMT and apoptosis in the progression of cancer. Our study demonstrated that silencing *KCNN4* elevated the expression of E-cadherin and Bax but reduced the expression of N-cadherin, Vimentin, Slug, Bcl-2 and Bcl-xl. These results indicated that KCNN4 may promote the EMT and inhibit apoptosis in PTC.

Our study had several shortcomings. First, we did not validate our results *in vivo*. Second, the crosstalk between the EMT and apoptosis in KCNN4-induced PTC progression requires further exploration. Finally, to increase the clinical significance of our findings, further experiments are needed to explore the effects of KCNN4 blockers on PTC.

In summary, we found that *KCNN4* was overexpressed in PTC and was a valuable diagnostic and prognostic marker. Silencing of *KCNN4* hindered the progression of PTC cell lines. Thus, KCNN4 is a potential therapeutic target in PTC.

## MATERIALS AND METHODS

### Bioinformatics

Public microarray datasets (GSE58689 [56], GSE9115 [57], GSE3678) including 44 PTC tissues and 29 nontumorous tissues were downloaded from the Gene Expression Omnibus (https://www.ncbi.nlm.nih.gov/geo/). Transcriptome sequencing data from 502 PTC samples and 58 normal thyroid tissues, along with the corresponding clinical data, were obtained from the thyroid cancer database of TCGA (https://www.cancer.gov/about-nci/organization/ccg/research/structural-genomics/tcga/studied-cancers/thyroid). R software (https://bioconductor.org/biocLite.R) was used to preprocess the data and investigate the differentially expressed genes. Differentially expressed genes in the microarray datasets were identified using the Limma package, with screening conditions of an adjusted p-value < 0.05 and a log2|fold change| ≥ 2. The heatmap was clustered using a Pearson correlation metric.

Pan-cancer analysis and immune infiltration evaluation were performed using the TIMER database (https://cistrome.shinyapps.io/timer/) [58]. Kaplan-Meier plots for disease-free survival were downloaded from the online database GEPIA (http://gepia.cancer-pku.cn) [59]. Single GSEA between the low and high *KCNN4* expression groups in TCGA was conducted using GSEA software (GSEA v3.0, http://www.broadinstitute.org/gsea) [60].

### Tissue samples

Forty-two matched pairs of PTC tissues and adjacent nontumorous tissues (patient age range: 16-72 years; male/female: 1/2) were dissected in the Department of Thyroid and Breast Surgery at The First Affiliated Hospital of Wenzhou Medical University (Wenzhou, Zhejiang, China) in 2018. After excision, the samples were instantly plunged into liquid nitrogen and frozen at -80 °C for long-term storage. Each specimen was histologically analyzed by three pathologists. Written informed consent was obtained from each patient. The clinical data were collected legally under protocols developed by the Committee for Medical Research Ethics of the First Affiliated Hospital of Wenzhou Medical University.

RNA sequencing data from 70 pairs of PTC tissues and matched adjacent nontumorous tissues were derived from our unpublished data.

### Cell culture

Human PTC cell lines (TPC-1, KTC-1 and BCPAP) were provided by Prof. Mingzhao Xing of the Johns Hopkins University School of Medicine (Baltimore, MD, USA). The normal human thyroid cell line (HTORI-3) was purchased from the Cell Bank of the Shanghai Chinese Academy of Sciences (Shanghai, China). The research resource identification number is CVCL_6298 for TPC-1, CVCL_6300 for KTC-1, CVCL_0153 for BCPAP and CVCL_4W02 for HTORI-3. The cells were cultured in RPMI 1640 medium containing 10% fetal bovine serum (Gibco, Invitrogen, Carlsbad, CA, USA) and 1% penicillin/streptomycin (Solarbio, Beijing, China) at 37 °C under 5% CO_2_.

### Cell transfection

SiRNAs (si-KCNN4 and si-NC) were designed and supplied by Gene Pharma (Shanghai, China). Cells were seeded in six-well plates one day before transfection (TPC-1, BCPAP: 60,000 cells per well; KTC-1: 80,000 cells per well). The siRNA was introduced into the cells using Lipofectamine RNAiMAX (Invitrogen, Grand Island, NY, USA) (siRNA:iMAX = 7.5 uL:3 uL). After seven hours, the medium was replaced. The cells were used after 48 hours for further experiments. The sequence of the *KCNN4*-targeted siRNA was as follows: KCNN4-homo-1481 sense 5’- GGGAACAAGUGAACUCCAUTT-3’/antisense 5’-AUGGAGUUCACUUGUUCCCTT-3’.

### RNA isolation, reverse transcription and qRT-PCR

Total RNA was extracted using TRIzol reagent (Thermo Fisher Scientific, Waltham, MA, USA), and reverse transcription was performed with a ReverTra Ace qPCR RT Kit (Toyobo, Osaka, Japan). The qRT-PCR was performed using a SYBR Premix Ex Taq II kit (RR820A, TaKaRa, Dalian, China) on an Applied Biosystems 7500 Real-Time PCR System. The mRNA expression relative to *GAPDH* expression was calculated using the 2^−ΔΔCt^ method. All measurements were performed according to standard protocols. The primers used in this study are shown in Supplementary Table 1 (Generay, Shanghai, China; Sangon, Shanghai, China).

### Cell proliferation assays

Cell viability was measured with a CCK-8 assay and a colony formation assay. TPC-1 cells (1,250 per well), KTC-1 cells (1,500 per well) and BCPAP cells (1,500 per well) were plated into 96-well plates. The cells were incubated with the CCK-8 reagent (Beyotime Biotechnology, Shanghai, China) for three hours (10 uL per well). Detection was performed at an absorbance of 450 nm on four consecutive days. For the colony formation assay, cells were seeded in six-well plates in the same numbers described above. At least five days later (> 50 cells/colony and > 30 colonies), colonies were fixed with 4% paraformaldehyde and stained with 0.1% crystal violet. Photos were taken under bright light. The experiments were carried out three times.

### Migration and invasion analysis

Cells were collected 48 hours after transfection and seeded (35,000 cells per well) into the upper chamber of a Transwell plate (#3422, Corning, NY, USA) with serum-free medium. The lower chamber was filled with 600 ul of medium containing 10% fetal bovine serum. After 22 hours, the cells that had migrated to the lower side of the membrane were fixed with 4% paraformaldehyde and stained with 0.1% crystal violet. Cells in five random visual fields from each chamber were imaged and counted under a microscope at ×20 magnification. A Matrigel invasion chamber (#354480, Corning Biocoat, Corning) was used with the same protocol to evaluate cellular invasion.

For the scratch wound assay, transfected cells were cultured under serum-free conditions at a density of 2×10^5^ cells per well in 24-well plates. A pipette tip was used to scratch the middle of each plate. Five fixed points were imaged at ×5 magnification before and after scratching. The migration rate (%) was calculated as (original wound area – 24-hour wound area) / original wound area × 100%. All experiments were performed at least three times.

### Flow cytometry analysis

Cell apoptosis was detected with an Annexin V-fluorescein isothiocyanate (FITC) apoptosis kit (#556547; Becton, Dickinson and Company, Franklin Lakes, NJ, USA) according to the manufacturer’s instructions. Cells were collected and washed three times with phosphate-buffered saline. Next, 500 uL of 1× binding buffer was used to resuspend the cells, and the cell suspension was dyed successively with Annexin V-FITC for 15 min and propidium iodide (PI) for 5 min in the dark. A flow cytometer (BD Biosciences Accuri C6; Becton, Dickinson and Company) and FlowJo software (FlowJo, Ashland, OR, USA) were used to analyze the cells. The Annexin V- / PI+, Annexin V+ / PI+, Annexin V+ / PI- and Annexin V- / PI- populations corresponded to necrotic cells (quadrant 1), late apoptotic cells (quadrant 2), early apoptotic cells (quadrant 3) and viable cells (quadrant 4), respectively.

### Western blotting

Total protein was extracted using radioimmunoprecipitation assay buffer (Solarbio) and protease inhibitors (Solarbio), and was measured with a bicinchoninic acid assay (Thermo Scientific, USA). The protein lysates were mixed with loading buffer and separated by 8-10% sodium dodecyl sulfate polyacrylamide gel electrophoresis (BioRad, Berkeley, CA, USA) at 80-120 V, and transferred to polyvinylidene difluoride membranes (EMD Millipore, Billerica, MA, USA) at 300 mA. After being blocked with 5% non-fat dried milk for two hours, the membranes were incubated with primary antibodies at 4 °C overnight. The antibodies used in this research are shown in Supplementary Table 2. Next, the blots were incubated with horseradish peroxidase-conjugated anti-rabbit or anti-mouse IgG (1:5000) secondary antibodies (Abcam, Cambridge, UK) at room temperature for two hours. Finally, proteins were visualized using an enhanced chemiluminescence (Thermo Scientific) detection system, and the images were analyzed with ImageJ software (NIH, Bethesda, MD, USA). Every experiment was repeated independently at least twice.

### Statistical analysis

Statistical analyses were performed using SPSS 22.0 software (IBM SPSS Inc, Chicago, IL, USA) and GraphPad Prism 8 software (GraphPad, San Diego, CA, USA). ROC curves were applied to evaluate the diagnostic efficacy of *KCNN4*. Kaplan-Meier curves were used to evaluate the impact of *KCNN4* expression on disease-free survival. Clinicopathological parameters were assessed using the χ2 test. The Shapiro-Wilk normality test (α = 0.05) was used to evaluate the data distribution. Normally distributed data were compared using Student’s t-test, while non-normally distributed data were analyzed using non-parametric tests (the Kruskal-Wallis test, Mann-Whitney test or Wilcoxon rank-sum test). Two-way analysis of variance was used for the CCK-8 assay. The results are presented as the mean ± standard deviation, and *p*-values < 0.05 were considered to be significant.

## Supplementary Material

Supplementary Tables
