# Supplementary material for: KCNN4 is a diagnostic and prognostic biomarker that promotes papillary thyroid cancer progression
Source: Aging (Albany NY). 2020 Aug 28;12(16):16437–56. doi: 10.18632/aging.103710 (PMC7485723; doi:10.18632/aging.103710)
Supplement: Supplementary Tables [file aging-12-103710-s001..pdf]

SUPPLEMENTARY TABLES

Supplementary Table 1. The primer sequences.

Gene	Sequence
KCNN4	F-GCAGAGGAGTAAGAAGGTGGAA
	R-TGGCAGGAACTGGCATTG
GAPDH	F-GTCTCCTCTGACTTCAACAGCG
	R-ACCACCCTGTTGCTGTAGCCAA
E-cadherin	F-AGTCACTGACACCAACGATAAT
	R-ATCGTTGTTCACTGGATTTGTG
N-cadherin	F-CGATAAGGATCAACCCCATACA
	R-TTCAAAGTCGATTGGTTTGACC
Vimentin	F-CCGACACTCCTACAAGATTTAGA
	R-CAAAGATTTATTGAAGGAGAACC
Slug	F-CTGTGACAAGGAATATGTGAGC
	R-CTAATGTGTCCTTGAAGCAACC
Bcl-2	F-GACTTCGCCGAGATGTCCAG
	R-GAACTCAAAGAAGGCCACAATC
Bax	F-CGAACTGGACAGTAACATGGAG
	R-CAGTTTGCTGGCAAAGTAGAAA

Abbreviations: F, forward sequence; R, reverse sequence.

Supplementary Table 2. List of antibodies.

Target	Source	Dilution	Company and catalog number
KCNN4	Rabbit	1:1000	Proteintech, 23271-1-AP
β-Actin	Mouse	1:5000	Proteintech, 60008-1-Ig
E-cadherin	Rabbit	1:5000	Proteintech, 20874-1-AP
N-cadherin	Rabbit	1:2000	Proteintech, 22018-1-AP
Vimentin	Rabbit	1:5000	Proteintech, 10366-1-AP
Slug	Rabbit	1:500	Proteintech, 12129-1-AP
Bcl-2	Rabbit	1:500	Abcam, ab59348
Bcl-xl	Rabbit	1:1000	Abcam, ab32370
Bax	Rabbit	1:4000	Proteintech, 50599-2-Ig
